# Genomic profiles of four novel cyanobacteria MAGs from Lake Vanda, Antarctica: insights into photosynthesis, cold tolerance, and the circadian clock

**DOI:** 10.3389/fmicb.2023.1330602

**Published:** 2024-01-11

**Authors:** Jessica Lumian, Christen Grettenberger, Anne D. Jungblut, Tyler J. Mackey, Ian Hawes, Eduardo Alatorre-Acevedo, Dawn Y. Sumner

**Affiliations:** ^1^Department of Earth and Planetary Sciences, Microbiology Graduate Group, University of California Davis, Davis, CA, United States; ^2^Department of Earth and Planetary Sciences, University of California Davis, Davis, CA, United States; ^3^Department of Environmental Toxicology, University of California Davis, Davis, CA, United States; ^4^Department of Sciences, The Natural History Museum, London, United Kingdom; ^5^Department of Earth and Planetary Sciences, University of New Mexico, Albuquerque, NM, United States; ^6^Coastal Marine Field Station, University of Waikato, Tauranga, New Zealand

**Keywords:** polar cyanobacteria, cryosphere, bioinformatics, genomics, photosynthesis, circadian clock

## Abstract

Cyanobacteria in polar environments face environmental challenges, including cold temperatures and extreme light seasonality with small diurnal variation, which has implications for polar circadian clocks. However, polar cyanobacteria remain underrepresented in available genomic data, and there are limited opportunities to study their genetic adaptations to these challenges. This paper presents four new Antarctic cyanobacteria metagenome-assembled genomes (MAGs) from microbial mats in Lake Vanda in the McMurdo Dry Valleys in Antarctica. The four MAGs were classified as *Leptolyngbya* sp. BulkMat.35, *Pseudanabaenaceae cyanobacterium* MP8IB2.15, *Microcoleus* sp. MP8IB2.171, and *Leptolyngbyaceae cyanobacterium* MP9P1.79. The MAGs contain 2.76 Mbp – 6.07 Mbp, and the bin completion ranges from 74.2–92.57%. Furthermore, the four cyanobacteria MAGs have average nucleotide identities (ANIs) under 90% with each other and under 77% with six existing polar cyanobacteria MAGs and genomes. This suggests that they are novel cyanobacteria and demonstrates that polar cyanobacteria genomes are underrepresented in reference databases and there is continued need for genome sequencing of polar cyanobacteria. Analyses of the four novel and six existing polar cyanobacteria MAGs and genomes demonstrate they have genes coding for various cold tolerance mechanisms and most standard circadian rhythm genes with the *Leptolyngbya* sp. BulkMat.35 and *Leptolyngbyaceae cyanobacterium* MP9P1.79 contained *kaiB3*, a divergent homolog of *kaiB*.

## 1 Introduction

Antarctic cyanobacteria form microbial mat communities that drive primary productivity in perennially ice-covered lakes (Hawes and Schwarz, 1999; Moorhead et al., 2005; Jungblut et al., 2016; Sumner et al., 2016; Dillon et al., 2020a). These microbial mats vary in texture and morphology among and within lakes, usually reflecting variations in community composition, environmental conditions, and ecological interactions (Hawes and Schwarz, 1999; Mackey et al., 2015; Jungblut et al., 2016; Sumner et al., 2016; Dillon et al., 2020a,b). The isolation of the lakes and their extreme environmental conditions limit the growth of microfauna within microbial mats, thus reducing grazing pressure. However, cyanobacteria must compete for limited environmental resources. Selection pressures include sparse nutrients in the oligotrophic environments and low irradiance under the thick ice cover. In addition, the available irradiance is dominated by short wavelengths, which selects organisms that are best suited to absorb and efficiently use available wavelengths (Howard-Williams et al., 1998; Vopel and Hawes, 2006). Mat organisms must also withstand cold lake water temperatures (−0.5 to 4°C), which slow metabolic rates, and polar seasons with extended winters with no photosynthetic activity. Despite these challenges, summer primary productivity can be high within Antarctic lakes (Hawes et al., 2001).

The seasonal light distribution is a significant challenge for phototrophic organisms in polar environments. Polar seasons dictate when photosynthesis is possible, and the McMurdo Dry Valleys have three months of constant light during summer and three months of darkness during winter. During the Antarctic winter, cyanobacteria must remain dormant or switch to another metabolic pathway in the absence of photosynthetic active radiation (PAR). The mechanisms for regulating these changes are unknown. In non-polar environments, circadian clocks facilitate diel changes in metabolisms by affecting gene expression, such as promoting gene expression for photosynthesis proteins during the day and nitrogen fixation proteins at night (Cohen and Golden, 2015; Liao and Rust, 2021). Because circadian clocks usually use photosynthesis to synchronize with environmental conditions on a diel cycle (Nayfach et al., 2020), the long periods of darkness and light in Antarctica may cause the circadian clock to lose synchronization with a 24-h cycle or may affect the ability of the clock to influence gene expression. Alternatively, polar cyanobacteria may contain additional genetic components to overcome these challenges. For example, some cyanobacteria possess diverged homologous versions of the standard *kaiABC* genes that encode the circadian clock. These *kaiA3B3C3* homologs may be linked to a metabolic switch in response to darkness (Aoki and Onai, 2009; Wiegard et al., 2020; Köbler et al., 2021), but they have not yet been identified in polar cyanobacteria.

Although cyanobacteria are responsible for a large portion of primary productivity in polar environments (Chrismas et al., 2018a), cyanobacteria from Antarctica and the Arctic are less well-studied than cyanobacteria in other regions (Anesio et al., 2009; Anesio and Laybourn-Parry, 2011; Chrismas et al., 2018b). This manuscript presents four novel cyanobacterial MAGs from pinnacle-shaped microbial mats in Lake Vanda, Antarctica, and compares them to six previously documented polar cyanobacteria [*Phormidium pseudopriestleyi* FRX01 (Lumian et al., 2021), *Candidatus* Aurora vandensis (Grettenberger et al., 2020), *Synechococcus* sp. CS-601 (SynAce01) (Tang et al., 2019), *Leptolyngbya* BC 1307 (Chrismas et al., 2018b), *Phormidesmis priestleyi* BC 1401 (Chrismas et al., 2016), and *Phormidesmis priestleyi* ULC007 (Lara et al., 2017)] with particular attention to genes relating to the unique challenges faced by polar cyanobacteria. Of particular interest is the extent to which genomic adaptations occur to deal with light harvesting, circadian rhythms, and cold tolerance in polar cyanobacteria. Photosynthesis and light harvesting genes were studied because the seasonal light availability of polar environments controls when cyanobacteria can perform photosynthesis. The *kaiABC* circadian clock genes have implications for the cycling of the circadian clock, which may be affected by periods of extended darkness or light, and *sasA*, *cikA*, and *rpaA* circadian clock genes affect gene expression. Cold stress genes were also analyzed because of their relevance to the functioning of cyanobacteria in polar conditions.

### 1.1 Lake Vanda site description

Lake Vanda is a perennially ice-covered lake in the Wright Valley, one of the McMurdo Dry Valleys of Southern Victoria Land, Antarctica. It receives water from the Onyx River, which flows ~30 km inland from the Lower Wright Glacier (Castendyk et al., 2016). The balance of this water flow with water loss from ablation determines the water level of the lake (Castendyk et al., 2016). There is a perennial ice cover 3.5–4.0 m thick, but ice around the shore melts during the summer to produce an open-water moat (Cutfield, 1974). Lake Vanda is well-illuminated compared to other McMurdo Dry Valley lakes, with 15–20% of photosynthetically active radiation (PAR) passing through the ice cover and a vertical extinction coefficient for downwelling PAR of 0.06 m^−1^ (Howard-Williams et al., 1989). Because ice transmits very little red light and water is transparent to blue-green light, the light spectrum is dominated by wavelengths shorter than 550 nm (Hawes and Schwarz, 2001). Thus, most irradiance available for photosynthesis in Lake Vanda is between 400 and 550 nm (Hawes and Schwarz, 2001), and these wavelengths are absorbed by the mat pigments (Sumner et al., 2016).

Well-illuminated conditions and a lack of erosion, burrowing, and grazing have allowed the growth of extensive microbial mats in Lake Vanda. The mats extend from just below the ice at the lake edge to more than 50 m water depth (Wharton et al., 1983; Hawes and Schwarz, 2001). The upper part of the water column in Lake Vanda is well mixed, resulting in similar environmental conditions for mats from 4–26 m depth except for irradiance (All depths are reported relative to lake level in 2013). The temperature of this upper convection zone is ~4°C (Castendyk et al., 2016). Mats in greater than 10 m water depths have morphologies ranging from <1-mm-tall tufts to centimeters tall pinnacles (Sumner et al., 2016). The largest pinnacles in the lake are up to ~30 cm tall, with cylindrical-shaped bases and cuspate tops. Photosynthetic activity in the mats has been demonstrated starting from just below the ice cover to at least 40 m depth (Hawes and Schwarz, 2001). Mats are laminated on an annual basis, providing an internal record of mat growth and evolution in shape (Hawes et al., 2001, 2013; Sumner et al., 2016). Pinnacles and prostrate mats between pinnacles have brown-colored surface layers with green and purple-pigmented areas in their interiors that are also photosynthetically active (Sumner et al., 2016). Below the photosynthetically active areas, the mat biomass is beige and lacks photosynthetic pigments (Hawes et al., 2013; Sumner et al., 2016).

Lake Vanda mats host communities similar to those in other McMurdo Dry Valley lakes (Zhang et al., 2015). 16S rRNA gene sequencing has shown that the bacterial and biomass is dominated by *Leptolyngbya*, *Phormidium, Tychonema* genotypes, pennate diatoms including *Navicula*, *Nitzchia*, *Caloneis*, and *Stauroneis*, and moss below 30 m depth (Kaspar et al., 1982; Love et al., 1983; Rankin et al., 2017).

## 2 Materials and methods

### 2.1 Photosynthetic active radiation (PAR) in Lake Vanda

We retrieved PAR data for November 1994 to January 2022 from Doran and Fountain (2016). The daily minimum, maximum, and mean irradiance values were calculated by averaging values from fifteen-minute intervals from each day. The values from each day were then averaged over the years. Analysis was done with the Python Pandas library (The Pandas Development Team, 2023).

### 2.2 Sample collection and DNA extraction

Scientific divers collected intact benthic microbial mats from 9 and 19 m lake depths in 2013 using utensils sterilized with alcohol wipes and brought samples to the surface in sterilized plastic containers. Pinnacles were dissected in a field lab based on the pigmentation of different zonations in the pinnacles (Sumner et al., 2016) using sterile technique and within 12 h of collection. Subsamples for genomic analysis were placed in Zymo Xpedition buffer (Zymo Research, Irvine, CA), and cells were lysed via bead beating. The stabilized samples were then frozen on dry ice and maintained frozen in the field. Upon leaving the field, samples were maintained at −20°C during storage and transport to UC Davis by the US Antarctic Program. Samples were stored at −80°C at UC Davis until DNA was extracted with the QuickDNA Fecal/Soil Microbe kit using the manufacturer’s instructions (Zymo Research, Irvine, CA, USA). The extracted DNAs were quantified using Qubit (Life Technologies, Carlsbad, CA, USA) and were concentrated via evaporation until the concentration was ≥10 ngL^−1^.

### 2.3 Illumina sequencing and bioinformatics processing

Sequencing was performed on 11 mat samples at the US Department of Energy Joint Genome Institute (JGI) using an Illumina HiSeq-2500 1 TB platform. An Illumina library was sequenced as 2 × 151 bp. BBDuk (v37.36) (Bushnell, 2014) removed common contaminants with the parameters removehuman = t, removedog = t, removecat = t, removemout = t, and removemicrobes = t. BBDuk was also used to trim reads containing adapter sequences and bases to the right of bases with a quality score of 0. It also removed reads containing 4 or more ‘N’ bases, with an average quality score less than 3, or had a minimum length less than 50 or 33% of the full read length.

Filtered and quality-controlled raw data was retrieved from IMG Gold from all metagenomes associated with JGI Gold Study ID Gs0127369. MEGAHIT v1.9.6 assembled metagenomes with a minimum contig length of 500 bp and a paired-end setting (Li et al., 2015). Bowtie2 v1.2.2 mapped reads back to the assembly (Langmead and Salzberg, 2012). A depth file was generated using jgi_summarize_bam_contig_depths from MetaBAT v2.12.1 and was used to bin contigs >2,500 bp (Kang et al., 2015). CheckM v1.0.7 was used to calculate the completeness and contamination of all bins (Parks et al., 2015). GTDB-tk v1.7.0 was used to classify bins with >70% completeness and < 5% contamination (Chaumeil et al., 2019), and only cyanobacterial bins were retained. Average nucleotide identity (ANI) between bins was calculated using the anvi-compute-genome-similarity command in Anvi’o v6.2 (Eren et al., 2015) using PyANI (Pritchard et al., 2016) and bins that shared >98% ANI were considered the same taxon. The bin with the lowest contamination and highest completeness was retained for each taxon. The Operon-mapper online web server was used to predict operons from the contigs in the MAGs (Taboada et al., 2018).

Previously sequenced cyanobacteria from Antarctica were downloaded from GenBank with the following accession numbers: *Phormidium pseudopriestleyi* FRX01 (ASM1731333v1) (Lumian et al., 2021), *cf. Aurora vandensis* (ASM1328554v1) (Grettenberger et al., 2020), *Synechococcus* (SynAce01; ASM188521v1) (Tang et al., 2019), *Leptolyngbya* BC 1307 (ASM228673v1) (Chrismas et al., 2018b), *Phormidesmis priestleyi* BC 1401 (ASM165019v1) (Chrismas et al., 2016) and *Phormidesmis priestleyi* ULC007 (ASM189592v1) (Lara et al., 2017). QUAST v5.0.2 was used to assess the quality of the assemblies (Gurevich et al., 2013). Annotation was performed with custom blast databases and KEGG ghostkoala annotations (Ogata et al., 1999; Camacho et al., 2009; Kanehisa et al., 2016). Average nucleotide identity (ANI) between the MAGs and previously sequenced cyanobacteria was calculated using the ANI calculator from the Kostas lab using the default parameters (Rodriguez-R and Konstantinidis, 2016). The code for this analysis is available here: https://github.com/jessicalumian/vanda_mags. Sequences for the MAGs are deposited in NCBI under accession numbers JARCMA000000000.1, JARCMB000000000.1, JARCMA000000000.1, and JARDMA000000000.1.

## 3 Results

### 3.1 PAR measurement in Lake Vanda

**Figure 1:**
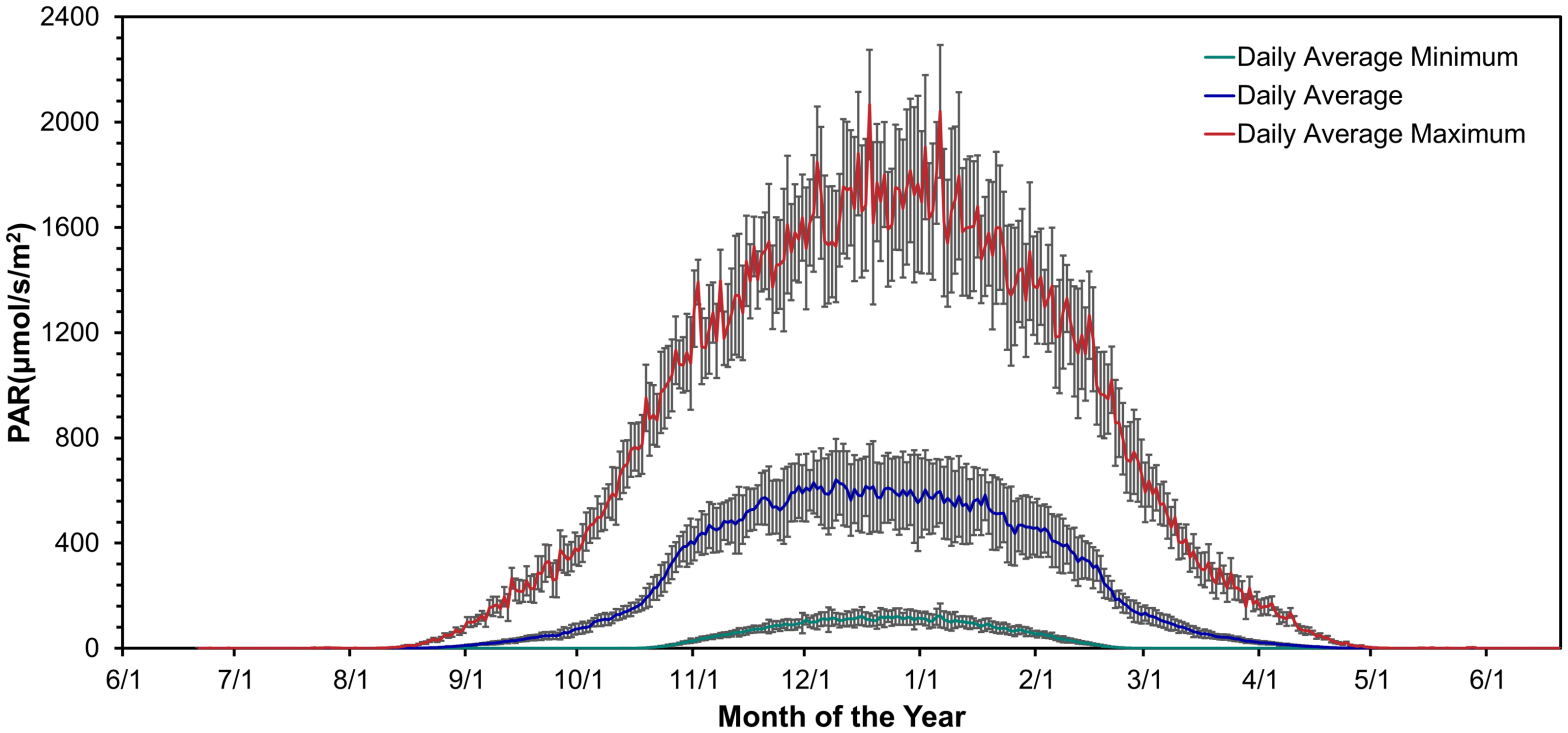
Daily mean PAR above the lake ice near Lake Vanda from November 1994 – January 2022. Data from Doran and Fountain (2016).

Irradiance was absent during winter from mid-April to mid-August (Figure 1; Doran and Fountain, 2016). Irradiance increased from September through March. Daily irradiance depended on cloud cover and weather conditions. The irradiance varied over a diel cycle from spring to fall. As fall and spring progressed, the days became shorter or longer toward constant darkness or continuous light but variable, respectively.

### 3.2 Sequencing statistics for bins and MAGs

The novel MAGs from Lake Vanda were classified as *Pseudanabaenaceae cyanobacterium* MP8IB2.15, *Microcoleus* sp. MP8IB2.171*, Leptolyngbya* sp. BulkMat.35, and *Leptolyngbyaceae cyanobacterium* MP9P1.79 (Supplementary Table S1). The MAGs contained between 2.76–6.07 Mbp and had GC content between 45.31–51.23% (Table 1). The median contig length is between 5,954 bp and 11,533 bp.

**Table 1:** Sequence statistics of MAG assigned to cyanobacteria in Lake Vanda microbial mats based on QUAST results.

Statistics	*Leptolyngbya* sp. BulkMat.35 (Vanda)	*Microcoleus* sp. MP8Ib2.171 (Vanda)	*Leptolyngbyaceae cyanobacterium* MP9P1.79 (Vanda)	*Pseudanabaenaceae cyanobacterium* MP8Ib2.15 (Vanda)	*Aurora vandensis* (Vanda)	*Synechococcus* SynAce01 (Ace Lake)	*Leptolyngbya* sp. BC 1307 (Hoare)	*Phormidesmis priestleyi* BC 1401 (Greenland Ice Shelf)	*Phormidesmis priestleyi* ULC 007 (Larsemann Hills)	*Phormidium pseudopriestleyi* FRX01 (Fryxell)
Longest contig (bp)	69,200	43,043	62,898	24,594	85,365	2,750,634	294,697	275,582	497,214	44,245
Total length (bp)	5,729,854	6,072,304	4.887,811	2,764,673	2,958,216	2,750,634	4,916,582	5,546,500	5,684,389	5,965,908
GC content (%)	51.23	45.47	50.03	45.31	55.41	63.92	52.94	49.17	48.62	47.43
N50	11,419	10,926	11,533	5,954	21,579	2,750,634	82,349	79,760	152,457	10,908
Number contigs ≥ 0 bp	654	671	552	505	202	1	222	213	118	678
Number contigs ≥ 1,000 bp	654	671	552	505	202	1	159	168	118	678
Number contigs ≥ 5,000 bp	406	476	349	232	164	1	97	121	79	458
Number contigs ≥ 10,000 bp	199	215	158	37	107	1	82	96	72	203
Number contigs ≥ 25,000 bp	24	19	25	0	32	1	49	62	55	20
Number contigs ≥ 50,000 bp	1	0	3	0	5	1	32	37	36	0

Three of the four Vanda MAGs were present in multiple metagenomic samples (Supplementary Table S1). The *Leptolyngbya* sp. BulkMat.35 MAG (JARCMC000000000.1) was from a prostrate mat located between pinnacles and was also present in eight samples from green and purple pigmented zones in small, medium, and large pinnacles. The *Microcoleus* sp. MP8IB2.171 MAG (JARCMA000000000.1) was from the beige interior of a different medium-sized pinnacle and was also present in seven samples from green, purple, and inner beige areas. The *Leptolyngbyaceae cyanobacterium* MP9P1.79 MAG (JARCMD000000000.1) was from a purple subsample of a medium-sized pinnacle and was also present in four subsamples from the green and beige interiors of medium pinnacles and a large pinnacle. The *Pseudanabaenaceae cyanobacterium* MP8IB2.15 MAG (JARCMB000000000.1) was only found in the beige interior of a medium-sized pinnacle (Supplementary Table S1).

### 3.3 Average nucleotide identity

In addition to the four MAGs presented here, five additional polar cyanobacteria for comparison include: *A. vandensis* from Lake Vanda, *P. pseudopriestleyi*, from Lake Fryxell, McMurdo Dry Valleys, *Synechococcus* SynAce01 from Ace Lake Vestfold Hills, *Leptolyngbya* BP 1307 from Lake Hoare, and *P. priestleyi* ULC 007 from Larsemann Hills. *Phormidesmis priestleyi* ULC 007 is from the Greenland Ice Sheet. The highest ANIs within all cyanobacteria in this study were 89–90% between the *Leptolyngbyaceae cyanobacterium* MP9P1.79, *Microcoleus* sp. MP8IB2.171, and *Leptolyngbya* sp. BulkMat.35 MAGs from Lake Vanda. *Phormidesmis pseudopriestleyi* BC 1401 and *Phormidium pseudopriestleyi* ULC 007 shared 83% ANI (Table 2). The four cyanobacteria MAGs from Lake Vanda were not closely related to the six comparison polar species. The Vanda MAGs shared less than 90% ANI with other cyanobacteria MAGs in this analysis.

**Table 2:** ANI matrix between novel Vanda MAGs and other polar cyanobacteria.

	*L. cyanobacterium* MP9P1.79	*Microcoleus* sp. MP8IB2.171	*P. pseudopriestleyi* FRX 01	*P. prestleyi* ULC 007	*P. priestleyi* BC 1401*	*Leptolyngbya* BC 1307	*Leptolyngbya* sp. BulkMat.35	*Synechococcus* SynAce01	*P. cyanobacterium* MP8IB2.15	*A. vandensis*
*L. cyanobacterium* MP9P1.79	100	90	-	75	76	-	89	-	-	-
*Microcoleus* sp. MP8IB2.171	90	100	74	75	74	-	-	-	-	-
*P. pseudopriestleyi* FRX 01	-	74	100	-	76	-	-	-	-	-
*P. prestleyi* ULC 007	75	75	-	100	83	75	75	-	-	-
*P. priestleyi* BC 1401*	76	74	76	83	100	77	77	-	-	-
*Leptolyngbya* BC 1307	-	-	-	75	77	100	73	-	-	-
*Leptolyngbya* sp. BulkMat.35	89	-	-	75	77	73	100	-	-	-
*Synechococcus* SynAce01	-	-	-	-	-	-	-	100	-	-
*P. cyanobacterium* MP8IB2.15	-	-	-	-	-	-	-	-	100	-
*A. vandensis*	-	-	-	-	-	-	-	-	-	100

ANI below 70% is not reported. Non-Antarctic cyanobacteria marked with *.

### 3.4 Genes associated with circadian rhythm in cyanobacteria

The posttranslational oscillator responsible for keeping time in cyanobacteria comprises proteins encoded by *kaiA*, *kaiB*, and *kaiC*. These genes are present in novel MAGs from Lake Vanda and the other polar cyanobacteria included in this analysis, except for *A. vandensis* and *Leptolyngbya* 1,307 MAGs (Table 3). Circadian rhythm genes are absent from all known members of the Gloeobacterales (Grettenberger, 2021). As *A. vandensis* shares a range of genes with and is phylogenetically related to gloeobacterial lineage, circadian rhythm genes are also likely absent in the *A. vandensis* genome. However, their absence in *Leptolyngbya* BC 1307 may be due to the incompleteness of the MAG. The *Leptolyngbya* sp. BulkMat.35 and the *Leptolyngbyaceae cyanobacterium* MP9P1.79 MAGs encode the standard *kaiB* gene as well as *kaiB3*. The *kaiB3* gene is a diverged homolog that may play a role in the metabolic switch from light to darkness (Wiegard et al., 2020). The main proteins involved in output signaling to affect gene expression are encoded by *sasA*, *cikA*, and *rpaA*. All the cyanobacteria MAGs and genomes included in this study contained multiple copies of these genes (Table 3).

**Table 3:** Copy number of genes encoding circadian clock genes in polar cyanobacteria.

Gene	*Leptolyngbya* sp. BulkMat.35 (L. Vanda)	*Microcoleus* sp. MP8IB2.171 (L. Vanda)	*L. cyanobacterium* MP9P1.79 (L. Vanda)	*P. cyanobacterium* MP8IB2.15 (L. Vanda)	*Aurora vandensis* (L. Vanda)	*SynAce01* (Ace Lake)	*Leptolyngbya* 1,307	*Phormidesmis priestleyi* 1,401	*Phormidesmis priestleyi* 007	*Phormidium pseudopriestleyi* FRX01 (L. Fryxell)
*kaiA*	1	1	1	1	0	1	1	1	1	1
*kaiB*	2	1	2	1	0	1	0	1	2	3
*kaiC*	1	1	1	1	0	1	0	1	1	2
*sasA*	4	3	0	1	>10	>10	>10	>10	>10	>10
*cikA*	3	4	4	4	1	3	2	0	0	2
*rpaA*	3	3	3	3	2	3	2	0	0	2

The *Leptolyngbya* sp. BulkMat.35, *Leptolyngbyaceae cyanobacterium* MP9P1.79, and *Pseudanabaenaceae cyanobacterium* MP8IB2.15 MAGs encoded *kaiA*, *B*, and *C* on the same operon according to Operon-mapper results. The *kaiB3* from the *Leptolyngbyaceae cyanobacterium MP9P1.79* MAG was on a different operon from the complete *kaiABC* group. The *Microcoleus* sp. MP8IB2.171 MAG contains *kaiA* and *kaiB* at the end of one contig, and an additional copy of *kaiA* on a different contig. *kaiC* was not identified on an operon, potentially because the genome is fragmented.

### 3.5 Photosynthesis genes in novel Lake Vanda MAGs

Phycoerythrocyanin, allophycocyanin, phycocyanin, and phycoerythrin are phycobilisome proteins with absorption peaks at 495 to 560, 650, 620, and 575 nm (Bogorad, 1975). All cyanobacteria from Lake Vanda contained the majority of genes necessary for synthesizing allophycocyanin and phycocyanin. Phycoerythrocyanin genes were present in *Microcoleus* sp. MP8IB2.171 and *Pseudanabaenaceae cyanobacterium* MP8IB2.15 but were absent in the *Leptolyngbya* sp. BulkMat.35 and *Leptolyngbyaceae cyanobacterium* MP9P1.79 MAGs. The pigment genes in the MAGs are consistent with the observed mat absorption of short wavelength irradiance (Sumner et al., 2016).

In addition to light-harvesting pigments, the MAGs from Lake Vanda contain many major key photosynthesis genes (Table 4). The presence and absence of phycobilisome and photosynthesis genes in these MAGs are consistent with other polar cyanobacteria and the majority of genes required for oxygenic photosynthesis (Shi and Falkowski, 2008) are present. The genes for the D1 and D2 proteins in Photosystem II are absent in most MAGs but this is likely due to MAG incompleteness.

**Table 4:** Presence of major photosynthesis genes in Lake Vanda MAGs.

Gene Category	Gene	*P. cyanobacterium* MP8IB2.15	*Microcoleus* sp. MP8IB2.171	*Leptolyngbya* sp. BulkMat.35	*L. cyanobacterium* MP9P1.79
Photosystem II D1/D2 cluster	*psbA*	No	No	Yes	No
	*psbB*	No	No	Yes	No
Chlorohyll apoprotein cp43	*psbC*	Yes	Yes	Yes	Yes
Chlorohyll apoprotein cp47	*psbB*	Yes	Yes	Yes	Yes
Cytochrome b559	*psbE*	Yes	Yes	Yes	Yes
	*psbF*	Yes	Yes	Yes	No
Oxygen Evolving Complex stabilization	*psbO*	No	No	Yes	Yes
	*psbP*	Yes	Yes	Yes	Yes
Cytochrome b6f	*petA*	No	Yes	Yes	Yes
	*petB*	Yes	Yes	Yes	Yes
	*petC*	No	Yes	Yes	Yes
	*petD*	Yes	Yes	Yes	Yes
	*petG*	No	No	No	No
	*petL*	No	No	No	No
	*petM*	No	No	Yes	No
	*petN*	Yes	No	No	No
Plastocyanin	*petE*	Yes	No	Yes	Yes
Cytochrome c6	*petJ*	No	Yes	Yes	Yes
Photosystem I Protein Dimer	*psaA*	Yes	Yes	Yes	Yes
	*psaB*	Yes	Yes	Yes	Yes
Ferredoxin	*petF*	Yes	Yes	Yes	Yes
Ferredoxin-NAPD+ reductase	*petH*	No	Yes	Yes	Yes
ATPase	*atpA*	Yes	No	Yes	Yes
	*atpB*	Yes	Yes	Yes	Yes
	*atpC*	Yes	No	Yes	Yes
	*atpD*	Yes	No	Yes	Yes
	*atpE*	Yes	Yes	Yes	Yes
	*atpF*	Yes	Yes	Yes	Yes
	*atpG*	Yes	Yes	Yes	Yes
	*atpH*	Yes	Yes	Yes	Yes

### 3.6 Cold tolerance genes

Cold shock genes allow cells to maintain normal functions, including transcription, translation, and energy production, at low temperatures, and these genes are often present in polar cyanobacteria (Table 5; Varin et al., 2012; Barria et al., 2013; Chrismas et al., 2016). The MAGs and genome in this study contain some, but not all of these genes. The gene for pyruvate dehydrogenase, *aceE*, is not present in any MAGs or genome in this study. Most encode *aceF*, the gene for the S-acyltransferase component of the pyruvate dehydrogenase complex, but it is absent from the *Leptolyngbya* sp. BulkMat.35 and *Pseudanabaenaceae cyanobacterium* MP8IB2.15 MAGs. The only cold tolerance gene relating to carbohydrate transport and metabolism, *otsA*, α,α-trehalose-phosphate synthase, is only present in the *Synechococcus* SynAce01 genome and *Leptolyngbya* BC1307 MAG. Several genes related to replication, recombination, and repair are in all the MAGs and genomes including, *deaD* (RNA helicase DeaD box), *dnaA* (replication initiator protein), and *gyrA* (DNA topoisomerase II). All except for the *Pseudanabaenaceae cyanobacterium* MP8IB2.15 MAG, encode *recA*, which is involved with DNA cleavage. The gene for a histone-like DNA binding protein, *hupB*, is absent from *Pseudanabaenaceae cyanobacterium* MP8IB2.15, *Leptolyngbyaceae cyanobacterium* MP9P1.79, *Microcoleus* sp. MP8IB2.171, and *A. vandensis* MAGs. A trigger factor protein involved in protein export, encoded by *tig*, is present in all MAGs and genomes except for the *Pseudanabaenaceae cyanobacterium* MP8IB2.15 MAG. Delta(12)-fatty-acid desaturase, encoded by *desA*, can change the degree of unsaturation of fatty acids at low temperatures (Wada et al., 1990), and it is present in all the MAGs and genomes. The transcription-related genes *rnr* (exoribonuclease II) and *nusA* (transcription termination) are present in all the MAGs and genomes, but *c*sp. genes (cold shock proteins) are absent from all of them. Some cold tolerance genes are involved in translation, ribosome structure, and biogenesis, such as *pnp*, *yfiA*, *rbfA*, and *infABC*. All MAGs and genomes include *pnp*, which codes for polyribonucleotide nucleotidyltransferase, and only the four cyanobacteria MAGs from Lake Vanda encode *yfiA*, which codes for ribosome-associated inhibitor A. The 30S ribosome-binding factor encoded by *rbfA* is absent from the *Leptolyngbyaceae cyanobacterium* MP9P1.79 MAG and *Synechococcus* SynAce01 genome. The translation initiation factors IF-1, IF-2, and IF-3 are encoded by *infABC* and are present in all MAGs and genomes in this study except the *Microcoleus* sp. MP8IB2.171 MAG, which is missing *infB*, and *P. pseudopriestleyi*, which lacks *infC*. There are two post-translational modification and chaperone cold shock genes: *dnaK* (chaperone protein DnaK) is present in all the MAGs and genomes but *dnaJ* (chaperone protein DnaJ) is not present in any MAGs from Vanda or in the *Synechococcus* SynAce01 genome.

**Table 5:** Cold tolerance genes present in the cyanobacteria genomes used in this study.

Gene category	Genes	*Leptolyngbya* sp. BulkMat.35 (L. Vanda)	*Microcoleus* sp. MP8IB2.171 (L. Vanda)	*L. cyanobacterium* MP9P1.79 (L. Vanda)	*P. cyanobacterium* MP8IB2.15 (L. Vanda)	*Aurora vandensis* (L. Vanda)
Energy production and conversion	*aceE*	No	No	No	No	No
	*aceF*	Yes	No	No	Yes	Yes
Carbohydrate transport and metabolism	*otsA*	No	No	No	No	No
Replication, recombination, and repair	*deaD*	Yes	Yes	Yes	Yes	Yes
	*recA*	Yes	Yes	Yes	No	Yes
	*dnaA*	Yes	Yes	Yes	Yes	Yes
	*gyrA*	Yes	Yes	Yes	Yes	Yes
	*hupB*	Yes	No	No	No	No
Cell cycle control, cell division, chromosome partitioning	*tig*	Yes	Yes	Yes	No	Yes
Lipid transport and metabolism	*desA*	Yes	Yes	Yes	Yes	Yes
Transcription	*rnr*	Yes	Yes	Yes	Yes	Yes
	*nusA*	Yes	Yes	Yes	Yes	Yes
	*csp*	No	No	No	No	No
Translation, ribosomal structure, and biogenesis	*pnp*	Yes	Yes	Yes	Yes	Yes
	*yfiA*	Yes	Yes	Yes	Yes	No
	*rbfA*	Yes	Yes	No	Yes	Yes
	*infA*	Yes	Yes	Yes	Yes	Yes
	*infB*	Yes	No	Yes	Yes	Yes
	*infC*	Yes	Yes	Yes	No	Yes
Post-translational modification, protein turnover, and chaperones	*dnaK*	Yes	Yes	Yes	Yes	Yes
	*dnaJ*	No	No	No	No	Yes

Gene category	Genes	*Synechococcus* SynAce01 (Ace Lake)	*Leptolyngbya* BC 1307	*P. priestleyi* BC 1401	*P. priestleyi* ULC 007	*P. pseudopriestleyi* FRX01 (L. Fryxell)
Energy production and conversion	*aceE*	No	No	No	No	No
	*aceF*	Yes	Yes	Yes	Yes	Yes
Carbohydrate transport and metabolism	*otsA*	Yes	Yes	No	No	No
Replication, recombination, and repair	*deaD*	Yes	Yes	Yes	Yes	Yes
	*recA*	Yes	Yes	Yes	Yes	Yes
	*dnaA*	Yes	Yes	Yes	Yes	Yes
	*gyrA*	Yes	Yes	Yes	Yes	Yes
	*hupB*	Yes	Yes	Yes	Yes	Yes
Cell cycle control, cell division, chromosome partitioning	*tig*	Yes	Yes	Yes	Yes	Yes
Lipid transport and metabolism	*desA*	Yes	Yes	Yes	Yes	Yes
Transcription	*rnr*	Yes	Yes	Yes	Yes	Yes
	*nusA*	Yes	Yes	Yes	Yes	Yes
	*csp*	No	No	No	No	No
Translation, ribosomal structure, and biogenesis	*pnp*	Yes	Yes	Yes	Yes	Yes
	*yfiA*	No	No	No	No	No
	*rbfA*	No	Yes	Yes	Yes	Yes
	*infA*	Yes	Yes	Yes	Yes	Yes
	*infB*	Yes	Yes	Yes	Yes	Yes
	*infC*	Yes	Yes	Yes	Yes	No
Post-translational modification, protein turnover, and chaperones	*dnaK*	Yes	Yes	Yes	Yes	Yes
	*dnaJ*	No	Yes	Yes	Yes	Yes

## 4 Discussion

The genes identified in the genomes of novel cyanobacteria from Lake Vanda provide insights into the features that allow cyanobacteria to thrive in polar freshwater environments. These organisms must employ survival mechanisms to deal with the challenges associated with polar environments including light availability and cold temperatures. Seasonal variations in light availability play a large role in controlling the metabolisms of photosynthetic organisms and are likely linked to the timing of the circadian clock. Cold temperatures slow the rates of biochemical processes and make cellular structures more brittle. Although the specific mechanisms of cold tolerance cannot be ascertained from genomic data alone, examining gene content provides a foundational understanding of how polar cyanobacteria propagate and thrive in challenging environments.

### 4.1 Polar light availability and cyanobacteria metabolism

In environments with diel cycles, photosynthesis is linked to synchronizing the circadian clock with environmental light (Swan et al., 2018). The circadian clock helps regulate metabolism on a daily cycle which is synchronized to light levels by the oxidation state of the quinone pool and ATP/ADP ratios (Cohen and Golden, 2015). In contrast, it is not known if or for how long polar organisms can maintain their circadian clocks on a ~ 24-h cycle in the absence of light in the winter, when light is continuous in the summer, or as light availability changes during the spring and fall. In winter, polar environments experience months-long dark seasons which prevent the circadian clocks from synchronizing with a 24-h day/night cycle. Synchronization fails in at least some organisms in the absence of light. For example, a study in *Drosophila* showed the circadian clocks of some species became arrhythmic during periods of constant darkness longer than 24 h (Bertolini et al., 2019). Additionally, cold temperatures affect the cycling of circadian clocks in cyanobacteria by nullifying kaiC oscillation due to Hopf bifurcation (Murayama et al., 2017). Therefore, circadian clocks in polar organisms may function differently than those in non-polar environments.

In the summer, from mid-October to mid-February, there is usually sufficient light for photosynthesis 24 h a day (Hawes et al., 2013; Figure 1). It is not clear how the circadian clock synchronizes with environmental conditions when cyanobacteria continuously photosynthesize under these light conditions (Hawes et al., 2013). The circadian clock may be able to maintain a 24-h cycle into the summer if the posttranslational oscillator encoded by *kaiABC* functions independently of the light environment. Lab experiments showed that the kaiC phosphorylation cycle in *Synechococcus elongatus* PCC 7942 persisted for at least 56 h in both continuous light and dark conditions, indicating that the posttranslational oscillator can run independently of environmental conditions in the short term (Nakajima et al., 2005; Tomita et al., 2005; Swan et al., 2018). Alternately, the amount of light in the summer changes over 24 h due to the height of the sun and topography. If differences in irradiance are large enough, the circadian clock may synchronize with a 24-h light cycle during the summer. For example, a small variation in daily light levels during summer months was sufficient to synchronize the circadian clock to environmental conditions in Arctic copepods (Hüppe et al., 2020). Similarly, photosynthetic rates in benthic mats were linearly proportional to photon flux in Lake Hoare (Hawes et al., 2014). Because photon flux has a diel cycle and also changes with cloud cover, it is possible that in summer, the cyanobacteria circadian clocks synchronize to photon flux changes, even in the absence of darkness.

Spring (mid-August to mid-October) and fall (mid-February to mid-April) represent transitional seasons with 24-h cycles including nighttime darkness. We hypothesize that polar cyanobacteria synchronize their circadian clocks in a similar way to those in non-polar environments during these transitional seasons. Periods of darkness increase during the fall and the clock can synchronize with the diel cycle until winter arrives. In the spring, light begins to appear, and cyanobacteria begin to photosynthesize. At this time, the clock likely begins to synchronize to daylight with the onset of photosynthesis providing the mechanism for synchronizing the circadian clock to the environment (Swan et al., 2018). Synchronization likely persists through spring conditions and may extend into the summer if diel light variations can provide the necessary synchronization.

Polar seasonality is predicted to affect gene expression because the output circadian proteins (e.g., *sasA*, *cikA*, and *rpaA*) are tied to the circadian clock (Swan et al., 2018) and affect gene expression. In the austral summer, class 1 genes, which are usually transcribed during the day would be transcribed whereas class 2 genes, which are usually transcribed at night, would not (Swan et al., 2018). For example, if polar cyanobacteria treat summertime conditions like one continuous day, then processes that normally occur at night, including respiration and nitrogen fixation may occur only minimally during the summer. During winter, genes associated with daytime conditions, such as photosynthesis genes, would not be expressed until light becomes available in the spring. However, these predictions depend on how the circadian clock functions during intervals of continuous darkness or light.

During the austral winter, polar cyanobacteria must remain dormant or use alternative energy sources, such as heterotrophy or fermentation, to survive in the absence of photosynthesis. Instead of photosynthesis, cyanobacteria may survive by metabolizing carbon storage molecules in their cells. Mats can grow and fix carbon during the summer and respire that fixed carbon during the winter without increasing biomass (Hawes and Schwarz, 2001). Cyanobacteria from Lake Vanda and the majority of other cyanobacteria included in this study have genes for storage molecules which may allow them to persist during periods of low metabolic activity; these include polyphosphates to store phosphates, cyanophycin, and phycobilins to store nitrogen, and glycogen to store carbon. Therefore, cyanobacteria may synthesize storage molecules during periods of active photosynthesis and growth in the summer. These molecules can be consumed during periods without photosynthesis including through the winter.

The seasonal metabolic activity of the cyanobacteria has implications for the ecology and geochemistry of polar microbial mats. Mats are most metabolically active during the summertime in Lake Vanda and nearby Lake Hoare (Hawes and Schwarz, 2001; Hawes et al., 2014). Shallow mats in Lake Vanda are estimated to fix 390 mg of carbon m^−2^ y^−1^ each summer (Hawes et al., 2013) and produce 120 μmol m^−2^ h^−1^ of O_2_ with PAR of 1 μmol quanta m^−2^ s^−1^ (Vopel and Hawes, 2006). Modeling of Lake Hoare mats predicted rates of O_2_ production with saturated irradiance to be between 543.8 and 537.5 μmol m^−2^ h^−1^, which is 22–23% higher than the observed O_2_ flux based on *in situ* measurements, suggesting that photosynthesis in the Lake Hoare mats is light limited (Vopel and Hawes, 2006). The organic component of the laminae in Lake Vanda microbial mats is linked to photosynthesis, and thicker organic laminae are due to increased rates of photosynthesis (Hawes et al., 2013; Sumner et al., 2016). Future work involving *in situ* transcriptomics and PAR measurements can be done to determine how closely the circadian clock is tied to photon flux and seasonality.

### 4.2 Polar cyanobacteria adaptations and cold tolerance genes

Polar cyanobacteria are primarily psychrotolerant rather than psychrophilic (Tang et al., 1997) and must be able to deal with cold stress challenges throughout the year. Based on ancestral state reconstruction, 20 cyanobacteria clades had ancestors from cold environments suggesting that they may be cold-adapted (Chrismas et al., 2015). However, these cyanobacteria do not share a unique suite of cold tolerance genes (Chrismas et al., 2018a,b). The lack of shared gene content between all cold-tolerant cyanobacteria suggests that polar cyanobacteria could use a variety of mechanisms to tolerate cold conditions, and these mechanisms could have a level of specificity across different environments through evolutionary history. Although polar cyanobacteria show higher growth rates at moderate temperatures, their tolerance to harsher conditions may allow them to outperform cyanobacteria that would normally be prevalent in moderate climates but cannot survive colder temperatures. Previous work has identified cyanobacteria inhabiting polar environments using 16S rRNA gene sequences (Jungblut et al., 2005; Chrismas et al., 2015; Zhang et al., 2015) but most lack genomes and nothing can be said about the metabolic potential of these cyanobacteria compared to those in more temperate environments. Therefore, more polar cyanobacteria genomes from a variety of environments are necessary to do comparative genomics and identify similarities and differences among polar cyanobacteria and closely related organisms in warmer environments to further elucidate signatures of cold-tolerant species (Chrismas et al., 2018b).

All of the polar cyanobacteria in this study contain cold tolerance genes, which are essential to their ability to thrive at permanently low temperatures in Antarctic lakes and other aquatic ecosystems. For example, the cyanobacteria in Lake Vanda experience temperatures around 4°C throughout the year. The ability of polar cyanobacteria to perform standard biochemical processes is essential for their survival, and all the organisms studied have genes for these processes. However, each cyanobacterium MAG or genome in this study is missing at least some genes that are associated with cold tolerance. Cyanobacteria from the cryosphere do not differ from relatives in warmer climates based on cold tolerance genes (Chrismas et al., 2016). The distribution of cold tolerance genes in our MAGs supports the hypothesis that these genes are present in most cyanobacteria and not specific to cold environments.

## 5 Conclusion

This work compares four new polar cyanobacteria MAGs to five polar cyanobacteria from different environments in Antarctica and one from the Greenland Ice Shelf. The four novel MAGs presented here are from Lake Vanda in the McMurdo Dry Valleys The genomic diversity demonstrated by these cyanobacteria indicates the need for more sequencing of cyanobacteria genomes both from established sampling environments and under sampled environments.

Based on the analysis of nine polar cyanobacterial MAGs and genomes most contain a complement of genes for cold tolerance and circadian clocks in addition to those for photosynthesis and other key metabolisms. Results demonstrate that circadian clock genes are commonly retained even though the organisms experience the extended darkness and continuous light of winter and summer, respectively. To build upon the foundational understanding laid out by analyzing genomic data, it would be highly advantageous to perform *in situ* experiments on the mats detailing the circadian clock function and gene expression of polar cyanobacteria in different lighting conditions.

## Data availability statement

The datasets presented in this study can be found in online repositories. The names of the repository/repositories and accession number(s) can be found in the article/Supplementary material. Sequences for the novel MAGs described in this paper are deposited in NCBI under accession numbers JARCMA000000000.1, JARCMB000000000.1, JARCMA000000000.1, and JARDMA000000000.1.

## Author contributions

JL: Conceptualization, Data curation, Formal analysis, Investigation, Methodology, Software, Validation, Visualization, Writing – original draft, Writing – review & editing. CG: Conceptualization, Funding acquisition, Investigation, Project administration, Software, Supervision, Writing – original draft, Writing – review & editing. AJ: Data curation, Investigation, Methodology, Project administration, Resources, Supervision, Writing – review & editing. TM: Data curation, Investigation, Project administration, Resources, Supervision, Writing – review & editing. IH: Data curation, Investigation, Resources, Supervision, Writing – review & editing. EA-A: Visualization, Writing – review & editing. DS: Conceptualization, Data curation, Investigation, Methodology, Project administration, Resources, Supervision, Writing – review & editing.
