# Supplementary material for: Genomic profiles of four novel cyanobacteria MAGs from Lake Vanda, Antarctica: insights into photosynthesis, cold tolerance, and the circadian clock
Source: Front Microbiol. 2024 Jan 11;14:1330602. doi: 10.3389/fmicb.2023.1330602 (PMC10812107; doi:10.3389/fmicb.2023.1330602)
Supplement: Supplementary file 1 [file Table_1.docx]

Supplementary Material

Genomic Profiles of Four Novel Cyanobacteria MAGs from Lake Vanda, Antarctica: Insights into Photosynthesis and the Circadian Clock

Jessica Lumian^1^, Christen Grettenberger^2,3*^, Anne D. Jungblut^4^, Tyler J. Mackey^5^, Ian Hawes^6^, Eduardo Alatorre-Acevedo^2^, Dawn Y. Sumner^2^

*** Correspondence:**Christen Grettenberger
clgrettenberger@ucdavis.edu

# Supplementary Table

**Table S1** Cyanobacteria bins over 70% complete from Lake Vanda mat samples. Bin completeness and contamination were determined by CheckM (42). Bolded rows were used for analysis.

| **Classification (GTDB-tk)** | **Mat Sample Characteristics** | **Bin Completeness** | **Bin Contamination** |
| --- | --- | --- | --- |
| *Leptolyngbya* | **Bulk mat between pinnacles (BulkMat_35; JARCMC000000000.1)** | **92.57%** | **0.63%** |
|  | Medium-sized pinnacle, green subsample (MP6G1_181) | 91.31% | 1.1% |
|  | Medium-sized pinnacle, green subsample (MP7G1_15) | 89.86% | 0.35% |
|  | Medium-sized pinnacle, green subsample (MP5G1_15) | 88.55% | 0.12% |
|  | Medium-sized pinnacle, purple subsample (MP7P2_86) | 85.81% | 0.47% |
|  | Medium-sized pinnacle, purple subsample (MP9P1_182) | 83.91% | 0.24% |
|  | Large-sized pinnacle (LV9_51) | 82.73% | 1.3% |
|  | Small-sized pinnacle, green subsample (SP4G1_162) | 78.25% | 0.24% |
| *Pseudanabaena* | **Medium-sized pinnacle, inner beige subsample (MP8IB2_15; JARCMB000000000.1)** | **74.2%** | **0.63%** |
| *Microcoleus* | **Medium-sized pinnacle, inner beige subsample (MP8IB2_171; JARCMA000000000.1)** | **88.88%** | **1.39%** |
|  | Medium-sized pinnacle, green subsample (MP5G1_130) | 87.72% | 1.09% |
|  | Medium-sized pinnacle, purple subsample (MP9P1_1) | 85.55% | 1.09% |
|  | Medium-sized pinnacle, green subsample (MP7G1_170) | 84.72% | 0% |
|  | Medium-sized pinnacle, purple subsample (MP7P2_141) | 81.66% | 2.19% |
|  | Medium-sized pinnacle, green subsample (MP6G1_116) | 79.5% | 0.55% |
|  | Medium-sized pinnacle, inner beige subsample (MP6IB1_67) | 75.48% | 0% |
| *Neosynechococcus* | **Medium-sized pinnacle, purple subsample (MP9P1_79; JARCMD000000000.1)** | **92.39%** | **0.24%** |
|  | Medium-sized pinnacle, green subsample (MP5G1_109) | 92.24% | 2.48% |
|  | Large-sized pinnacle (LV9_51) | 91.29% | 1.65% |
|  | Medium-sized pinnacle, inner beige subsample (MP6IB_67) | 75.48% | 0% |

**Table S2** Photosynthesis Genes in MAGs. Yes indicates that all genes present. Most indicates that 2/3 or more genes present. Some indicates 1/3 or more genes present. No indicates that no genes present.

| **Gene Group** | ***Leptolyngbya* sp. BulkMat.35 (L. Vanda)** | ***Microcoleus* sp. MP8IB2.171 (L. Vanda)** | ***L. cyanobacterium* MP9P1.79 (L. Vanda)** | ***P. cyanobacterium* MP8IB2.15 (L. Vanda)** | ***A. vandensis* (L. Vanda)** |
| --- | --- | --- | --- | --- | --- |
| Allophycocyanin | Yes | Yes | Most | Yes | Yes |
| Phycocyanin / Phycoerythrocyanin | Most | Yes | Yes | Yes | Most |
| Phycoerythrin | No | Most | No | Some | No |
| Photosystem II | Most | Most | Some | Some | Some |
| Photosystem I | Some | Some | Some | Some | Some |
| Cytochrome b6f | Some | Some | Most | Some | Some |
| Photosynthetic electron transport | Yes | Most | Yes | Some | Yes |
| F-type ATPase | Yes | Most | Yes | Yes | Most |

| **Gene Group** | ***SynAce* (Ace Lake)** | ***Leptolyngbya* 1307** | ***P. priestleyi* BC1401** | ***P. priestleyi ULC 007*** | ***P. pseudopriestleyi* FRX01 (L. Fryxell)** |
| --- | --- | --- | --- | --- | --- |
| Allophycocyanin | Yes | Yes | Most | Yes | Yes |
| Phycocyanin / Phycoerythrocyanin | Most | Yes | Yes | Yes | Most |
| Phycoerythrin | Yes | Most | Most | Most | Most |
| Photosystem II | Some | Most | Most | Most | Some |
| Photosystem I | Some | Some | Most | Most | Some |
| Cytochrome b6f | Some | Some | Most | Most | Some |
| Photosynthetic electron transport | Yes | Yes | Yes | Yes | Most |
| F-type ATPase | Yes | Yes | Yes | Yes | Yes |
